# Comparative Study of Feed Form Effects on Productive Performance, Egg Quality and Nutrient Utilization in Laying Hens

**DOI:** 10.3390/ani15233420

**Published:** 2025-11-27

**Authors:** Jae Hong Park, Hyesuk Kim, In Ho Kim

**Affiliations:** 1 Department of Animal Biotechnology, Dankook University, Cheonan 31116, Republic of Korea; parkjh@dankook.ac.kr; 2 Smart Animal Bio Institute, Dankook University, Cheonan 31116, Republic of Korea; 3 Department of Research & Development of Central Institute, Daehan Feed Company, Incheon 22693, Republic of Korea; dastcau@naver.com

**Keywords:** egg quality, feed form, laying hens, nutrient digestibility, productive performance

## Abstract

A 16-week feeding trial was conducted to evaluate the effects of different feed forms on the productivity, egg quality, nutrient digestibility, and organ development of laying hens. A total of 252 Lohmann Brown laying hens, 20 weeks of age, were randomly assigned to one of three dietary treatments: mash, pellet, or crumble. Each treatment consisted of seven replicates with 12 hens per replicate. All diets were formulated primarily with corn and soybean meal to provide 2801 kcal/kg of metabolizable energy and 17.99% crude protein. Productive performance (egg production, egg weight, feed intake, egg mass, and feed conversion ratio), egg quality traits (haugh unit, eggshell strength, and eggshell thickness), apparent nutrient digestibility (dry matter, crude protein, calcium, and phosphorus), and relative organ weights (crop, gizzard, and abdominal fat) were measured. No significant differences (*p* > 0.05) were observed among the feed form treatments for productive performance, egg quality, nutrient digestibility, or organ development. These results indicate that feed form (mash, pellet, or crumble) does not significantly affect performance, egg quality, nutrient utilization, or organ development in Lohmann Brown laying hens under the conditions of this study.

## Simple Summary

Feed form is known to influence feed intake and nutrient utilization in poultry. However, its impact on laying hens is still unclear. In this study, we compared three feed forms—mash, pellet, and crumble—in Lohmann Brown laying hens over a 16-week period. The results showed that feed form did not affect egg production, egg quality, nutrient digestibility, or organ development. These findings indicate that laying hens can maintain normal productivity and health regardless of whether they are fed mash, pellet, or crumble diets, allowing flexibility for producers when choosing feed form based on cost or management preferences.

## 1. Introduction

Feed form is a critical factor influencing feed intake behavior, nutrient digestibility, and overall performance in poultry production. In laying hens, where optimal egg production and feed efficiency are essential, the physical structure of feed can significantly affect both physiological responses and productive outcomes. Among the commonly used feed forms—mash, pellet, and crumble—each possesses distinct physical and functional characteristics that may influence nutrient utilization and performance parameters in different ways [1,2].

Mash feed, composed of finely ground and unprocessed ingredients, is widely used due to its simplicity and low production cost. However, its fine particle size may encourage selective feeding and result in less uniform nutrient intake [3]. Pelleted feed, produced by steam-conditioning and compressing mash through a die, has been shown to enhance feed intake, nutrient digestibility, and growth performance in broilers. It may confer similar benefits in laying hens by increasing feed density and reducing feed wastage [2]. Crumble feed, made by breaking pellets into smaller granules, is often used as a transitional form and may improve palatability and handling characteristics, especially for younger or more selective birds [4].

While numerous studies have investigated the effects of feed form in broilers, relatively few have focused on laying hens, particularly in relation to long-term performance, egg production, egg quality traits, and nutrient digestibility. Given the unique physiological and metabolic profiles of layers compared to meat-type birds, it is important to evaluate how feed form impacts performance across the laying cycle.

Therefore, the objective of this study was to evaluate and compare the effects of mash, pellet, and crumble diets on laying performance, egg quality, nutrient digestibility, and organ weights in commercial laying hens. The findings aim to provide evidence-based recommendations for optimal feed form selection to improve productivity and feed efficiency in layer production systems.

## 2. Materials and Methods

Ethical approval for the animal procedures used in this study was granted by the Institutional Animal Care and Use Committee of Dankook University, Korea (Approval No. DK-1-2417_2).

### 2.1. Experimental Design, Animals and Managements

A total of 252 Lohmann Brown laying hens at 20 weeks of age (average body weight: 1740 ± 18 g) were used in this study. Before random allocation, birds were weighed individually, and those with similar body weights were evenly distributed among treatments. Randomization was performed using a random number generator, and cage positions were balanced across treatments to minimize environmental bias. The experiment followed a completely randomized design with three dietary treatments, each consisting of seven replicates with twelve birds per replicate. The feeding trial lasted for 16 weeks.

Experimental diets were provided in three different physical forms: mash, pellet, and crumble. The particle size distribution of each feed was analyzed using a Ro-Tap sieve shaker (Model RX-29, W.S. Tyler, Mentor, OH, USA) equipped with a standard set of sieves (4.75–0.25 mm). The geometric mean diameter (GMD) and geometric standard deviation (GSD) were calculated according to the ASAE S319.4 method. The GMD and GSD values of mash, pellet, and crumble diets were 1.4 mm (1.95), 3.5 mm (1.24), and 1.8 mm (1.58), respectively. Pelleted diets were processed using a ring-die pellet mill with a 4 mm die hole and 6 mm length. The mash feed was conditioned with steam at 80 °C for 30 s before pelleting and cooled at ambient temperature (25 °C) for 20 min before crumbling. Crumble feed was prepared by crushing the cooled pellets using a crumbler to achieve a particle size of approximately 3 mm.

All experimental diets were formulated primarily with corn and soybean meal to provide 2801 kcal/kg of metabolizable energy and 17.99% crude protein (Table 1). The nutrient composition was designed to meet or exceed the recommendations of the NRC [5] and the Korean Feeding Standard for Poultry [6]. Throughout the experimental period, hens were housed in 3-tier cages equipped with nipple drinkers, and they had ad libitum access to feed and water. A photoperiod of 16 h of light and 8 h of darkness (16L:8D) was maintained. The average ambient temperature in the poultry house was controlled at 22 °C, and the relative humidity was maintained at approximately 60%.

### 2.2. Laying Performance

The number of eggs laid, feed residues, and egg weights were recorded daily for each treatment group throughout the experimental period. Based on the recorded data, hen-day egg production rate, feed intake, and feed conversion ratio (FCR) were calculated. The hen–day egg production rate was calculated by dividing the number of eggs laid by the number of hens. Feed intake was determined by subtracting the feed residues from the total feed supplied. The FCR was calculated by dividing the feed intake by the egg mass.

### 2.3. Egg Quality

Egg quality characteristics were evaluated at 4-week intervals (weeks 4, 8, 12, and 16) after the start of the experiment. On the final day of each interval, 30 eggs were randomly collected from each treatment group for analysis. Haugh unit, eggshell thickness, and eggshell strength were measured using a digital egg tester (Digital Egg Tester, DET-6000, NABEL Co., Kyoto, Kyoto-fu, Japan). The Haugh unit was calculated based on egg weight (W) and albumen height (H) using the following formula:

[100 × log (H − (1.7 × W^0.37^) + 7.57)].

### 2.4. Nutrient Digestibility

To determine the apparent digestibility of dry matter, crude protein, calcium, and phosphorus, 0.2% chromium oxide (Cr_2_O_3_) was added to the experimental diets as an indigestible marker during the final week of the trial. After feeding the chromium-supplemented diets for 7 days, apparent digestibility was assessed using the indirect marker method. Feed and fecal samples were dried at 60 °C for 72 h and subsequently ground for analysis. Crude protein (CP) was determined using the Kjeldahl method (AOAC 984.13). Nitrogen (N) concentration was measured following acid digestion and distillation, and CP was calculated as N × 6.25. Calcium and phosphorus were determined after dry-ashing samples at 550 °C (AOAC 942.05); the resulting ash was solubilized in HCl. Calcium concentration was analyzed using atomic absorption spectrophotometry (AOAC 985.01), and phosphorus concentration was measured colorimetrically using the molybdenum-vanadate method (AOAC 965.17). Chromium concentrations in feed and fecal samples were analyzed with an atomic absorption spectrophotometer (PerkinElmer AAnalyst 400, PerkinElmer, Waltham, MA, USA). Apparent digestibility (%) was calculated using the following formula:

100 − [100 × (Cr in feed/Cr in feces) × (Nutrient in feces/Nutrient in feed)].

### 2.5. Organ Weights and Abdominal Fat

At the end of the experimental period (16 weeks), fourteen birds were randomly selected from each treatment group. After stunning with CO_2_ gas and slaughtering, the weights of the crop, gizzard, and abdominal fat were measured and expressed as a percentage of the live body weight.

### 2.6. Statistical Analyses

The data were analyzed using a randomized block design followed by one-way ANOVA. When significant differences were observed, Tukey’s HSD test was performed for pairwise comparisons among the three treatment groups. Statistical significance was considered at *p* < 0.05. A post hoc power analysis was performed using the observed variance and sample size for the main performance parameters (egg production, feed intake, and feed conversion ratio). The calculated power (1–β) values ranged from 0.82 to 0.89, confirming that the experimental design provided sufficient sensitivity to detect biologically relevant differences among treatments.

## 3. Results

### 3.1. Laying Performance

The results of laying performance in laying hens fed different feed forms over a 16-week period are presented in Table 2. During weeks 1 to 4, no significant differences were observed in egg production among the dietary treatments (mash, pellet, and crumble). Similarly, no differences were detected in egg weight, feed intake, egg mass, or feed conversion ratio during the same period. This pattern was consistently observed during the subsequent evaluation periods (weeks 1–4, 5–8, 9–12, and 13–16), suggesting that variations in feed processing form did not affect laying performance, including egg production, egg weight, feed intake, egg mass, and feed conversion ratio in laying hens.

### 3.2. Egg Quality

No significant differences were observed among the dietary treatments (mash, pellet, and crumble) in Haugh unit, eggshell strength, or eggshell thickness at weeks 4, 8, 12, and 16, indicating that the form of feed processing did not influence these egg quality parameters in laying hens (Table 3).

### 3.3. Nutrient Digestibility

At week 16, apparent digestibility of dry matter, crude protein, calcium, and phosphorus did not differ significantly among the dietary treatments (mash, pellet, and crumble) (Table 4). These results suggested that feed form had no effect on nutrient digestibility in laying hens.

### 3.4. Organ Weights and Abdominal Fat

At week 16, there were no significant differences in the relative weights of the crop, gizzard, or abdominal fat among the different dietary treatments (mash, pellet, and crumble). These results suggest that feed form had no substantial effect on organ development or fat deposition in laying hens (Table 5).

## 4. Discussion

This study investigated how different physical feed forms—mash, pellet, and crumble—affect various aspects of laying hen performance, including production efficiency, egg quality, nutrient digestibility, and organ development. Despite previous findings suggesting performance benefits from processed feeds such as pellets and crumbles over mash diets in poultry [3,7,8], our results revealed no significant differences among treatments in laying performance parameters such as egg production rate, feed intake, or feed conversion ratio. Although pelleted and crumbled diets are known to enhance nutrient digestibility, reduce feed wastage, and increase energy availability—mainly due to their improved physical uniformity and reduced feed selection [1,2,9]—these advantages are typically more relevant to broilers, which have higher growth rates and nutrient demands. In contrast, laying hens exhibit relatively stable metabolic requirements and slower growth, possibly diminishing their responsiveness to feed processing. Furthermore, their more selective feeding behavior and slower feed passage rate may enable efficient nutrient utilization even with mash diets [10]. When mash is appropriately ground and well-mixed, differences in nutrient accessibility between mash and processed feeds may become negligible. Thus, while feed processing into pellets or crumbles may benefit broiler performance, its impact on laying hens appears limited under the present experimental conditions. The absence of significant performance differences among feed forms may be attributed to the use of diets with commercially typical particle sizes and balanced nutrient composition. When feed particle size is maintained within the optimal commercial range, differences in feed form (mash, pellet, or crumble) are less likely to cause notable variation in feed intake or nutrient digestibility. From an economic perspective, pelleting and crumbling processes increase manufacturing costs and energy consumption compared with mash production. Therefore, when performance remains unaffected, mash feed may offer a more cost-effective option for commercial producers. However, pelleted or crumbled feeds can still provide practical advantages in automated feeding systems and under conditions where feed wastage or segregation occurs. Overall, the present findings suggest that feed form selection may be guided primarily by feed mill efficiency and management convenience, rather than by concerns about productivity differences among forms.

Regarding egg quality, no significant treatment effects were observed for Haugh units, eggshell thickness, or shell strength. Although processed feeds provide uniform particle size and mixing homogeneity—factors that could theoretically reduce selective feeding and enhance balanced nutrient intake—the expected improvements in egg quality were not evident. This is in line with previous studies suggesting that the influence of feed form on egg quality is minimal when diets are nutritionally balanced [8,11]. One possible explanation is that laying hens, with their mature digestive systems and steady nutrient needs, may be less sensitive to physical differences in feed. Moreover, egg quality is shaped by multiple interacting factors, including calcium source, dietary formulation, particle size distribution, and genetic background [8,12,13]. The lack of significant variation in our study may reflect the consistency of diet composition across all groups, particularly in calcium and other critical nutrients. These findings imply that under conditions of well-balanced nutrition, physical feed structure alone may not significantly alter egg quality outcomes.

In terms of nutrient digestibility, no significant differences were observed in the apparent digestibility of dry matter, crude protein, calcium, or phosphorus. This indicates that feed form had a limited effect on nutrient utilization in laying hens under our experimental conditions. While improved digestibility with pelleted or crumbled feeds has been widely reported in broilers—owing to better homogeneity, reduced anti-nutritional factors, and increased starch gelatinization during pelleting [1,2]—such benefits were not apparent here. This may be due to the use of isonutrient diets and the more stable feeding patterns typical of layers. Additionally, the relatively well-developed gizzard in laying hens likely enhances the mechanical processing of mash feed, thereby reducing any comparative advantage of processed forms [14]. Our findings support the notion that when feed particle size and nutrient distribution are appropriately managed, diet form may exert little influence on nutrient absorption efficiency.

No differences were detected in the relative weights of the crop, gizzard, or abdominal fat pad among the dietary groups. Previous studies have suggested that unprocessed diets like mash, which require more grinding, stimulate gizzard development, while pelleted or crumbled feeds may reduce gizzard activity due to their compact structure and lower grinding requirements [1,15,16,17]. However, in the present study, the comparable organ weights across treatments suggest that all diets were adequately balanced in terms of fiber content and structural integrity to support normal digestive function. Similarly, the absence of treatment effects on abdominal fat pad weight indicates that feed form did not alter energy metabolism or storage, likely due to the isocaloric and isonitrogenous nature of the diets. Given the 16-week duration of the study, which allowed sufficient time for cumulative physiological effects to emerge, the results further reinforce the limited influence of feed form on visceral development and fat deposition in laying hens.

Overall, our findings indicate that when nutrient content, particle size, and feed formulation are carefully controlled, the physical structure of feed—whether mash, pellet, or crumble—has limited influence on performance, nutrient digestibility, egg quality, or organ development in laying hens.

## 5. Conclusions

In this 16-week feeding trial, feed form (mash, pellet, or crumble) did not significantly affect performance, egg quality, nutrient digestibility, or organ development in Lohmann Brown laying hens. These results indicate that under the tested conditions and feed particle sizes, feed form exerted minimal influence on hen productivity and nutrient utilization. Further studies including a wider range of feed diameters and physiological parameters are recommended.

## Figures and Tables

**Table 1:** Basal diet composition used in the experimental feeding trial (as fed-basis).

Ingredients	%
Corn	54.456
Soybean meal	22.900
Dried distillers’ grains with solubles	10.000
Limestone	9.800
Palm kernel meal	1.600
Tallow	0.500
Salt	0.250
Monodicalcium phosphate	0.100
Vitamin mix ^1^	0.100
Mineral mix ^2^	0.100
Choline	0.100
Methionine	0.094
Total	100.00
Calculated value	
Metabolizable energy, kcal/kg	2801
Crude Protein, %	17.99
Methionine + Cystine, %	0.74
Calcium, %	3.84
Available Phosphorus, %	0.41
Crude Fat, %	3.24
Crude Fiber, %	3.37
Crude Ash, %	13.08

^1^ Provided per kg of diet: vitamin A, 8000 IU; vitamin D3, 3300 IU; vitamin E, 20 IU; vitamin K3, 2.5 mg; vitamin B1, 2.5 mg; vitamin B2, 5.5 mg; vitamin B6, 4 mg; vitamin B12, 23 mg; biotin, 75 mg; folic acid, 0.9 mg; niacin, 30 mg; D-calcium pantothenate, 8 mg. ^2^ Provided per kg of diet: Fe, 40 mg as ferrous sulfate; Cu, 8m g as copper sulfate; Mn, 90 mg as manganese oxide; Zn, 80 mg as zinc oxide; 1.2 mg as potassium iodide; Se, 0.22 mg as sodium selenite.

**Table 2:** Effects of different feed forms on egg production parameters in laying hens.

	Feed Form			SEM ^1^	*p*-Value
	Mash	Pellet	Crumble		
Weeks 1–4					
Egg production, %	91.2	91.1	91.6	1.41	0.9886
Egg weight, g	55.7	55.8	56.1	0.52	0.9390
ADFI ^2^, g	104.5	104.7	105.0	1.34	0.9906
Egg mass	50.8	50.9	51.4	1.26	0.9892
FCR ^3^	2.065	2.068	2.050	0.03	0.9672
Weeks 5–8					
Egg production, %	96.3	96.7	97.3	1.59	0.7549
Egg weight, g	59.3	59.9	60.0	0.27	0.4253
ADFI, g	112.1	113.6	113.0	0.90	0.6773
Egg mass	57.1	58.0	58.4	1.30	0.6999
FCR	1.968	1.963	1.939	0.04	0.1358
Weeks 9–12					
Egg production, %	96.0	96.7	97.5	1.81	0.7005
Egg weight, g	61.4	62.1	62.3	0.17	0.3013
ADFI, g	113.2	114.6	114.0	1.14	0.6403
Egg mass	58.9	60.1	60.7	0.23	0.6245
FCR	1.925	1.913	1.883	0.04	0.4496
Weeks 13–16					
Egg production, %	95.2	96.3	97.0	0.23	0.6667
Egg weight, g	62.6	63.1	63.2	0.12	0.5214
ADFI, g	113.5	114.5	114.2	0.14	0.7063
Egg mass	59.6	60.7	61.3	0.22	0.6063
FCR	1.908	1.890	1.865	0.05	0.6225

^1^ standard error of means; ^2^ average daily feed intake; ^3^ feed conversion ratio. Values are presented as means ± SEM (*n* = 7 replicates per treatment; each replicate = 12 birds).

**Table 3:** Effects of different feed forms on egg quality in laying hens.

	Feed Form			SEM ^1^	*p*-Value
	Mash	Pellet	Crumble		
Week 4					
Haugh unit	95.6	95.8	95.9	0.46	0.6422
Eggshell strength, kg/cm^2^	4.29	4.28	4.31	0.15	0.8230
Eggshell thickness, mm^−2^	39.6	39.2	39.7	0.42	0.4530
Week 8					
Haugh unit	94.0	94.3	94.5	0.45	0.4641
Eggshell strength, kg/cm^2^	4.24	4.22	4.27	0.25	0.7454
Eggshell thickness, mm^−2^	40.2	39.7	40.4	0.46	0.1592
Week 12					
Haugh unit	91.8	92.2	92.4	0.74	0.6473
Eggshell strength, kg/cm^2^	4.18	4.14	4.20	0.17	0.7780
Eggshell thickness, mm^−2^	39.7	39.3	39.8	0.62	0.3255
Week 16					
Haugh unit	89.8	90.1	90.3	0.93	0.7170
Eggshell strength, kg/cm^2^	4.09	40.6	4.13	0.23	0.7194
Eggshell thickness, mm^−2^	39.5	38.9	38.0	0.61	0.2837

^1^ standard error of means. Values are presented as means ± SEM (*n* = 30 eggs per treatment at each sampling time).

**Table 4:** Effects of different feed forms on nutrient digestibility in laying hens.

	Feed Form			SEM ^1^	*p*-Value
	Mash	Pellet	Crumble		
Week 16					
Dry matter, %	83.13	83.21	83.42	0.85	0.8350
Crude protein, %	58.72	58.93	59.28	0.77	0.7022
Calcium, %	56.06	55.73	56.37	0.71	0.5314
Phosphorus, %	46.34	45.93	46.67	0.46	0.2412

^1^ standard error of means. Values are presented as means ± SEM (*n* = 14 birds per treatment).

**Table 5:** Effects of different feed forms on organ weight in laying hens.

	Feed Form			SEM ^1^	*p*-Value
	Mash	Pellet	Crumble		
Week 16					
Crop, %	0.76	0.79	0.79	0.03	0.8242
Gizzard, %	1.75	1.77	1.79	0.04	0.9198
Abdominal fat, %	1.19	1.20	1.19	0.03	0.6983

^1^ standard error of means. Values are presented as means ± SEM (*n* = 14 birds per treatment).

## Data Availability

The authors confirm that the data supporting the findings of this study are available upon the request from the corresponding author.
